# Intra-articular injection of synthetic microRNA-210 accelerates avascular meniscal healing in rat medial meniscal injured model

**DOI:** 10.1186/s13075-014-0488-y

**Published:** 2014-11-28

**Authors:** Yoshitaka Kawanishi, Tomoyuki Nakasa, Takeshi Shoji, Michio Hamanishi, Ryo Shimizu, Naosuke Kamei, Muhammad Andry Usman, Mitsuo Ochi

**Affiliations:** Department of Orthopaedics Surgery, Integrated Health Sciences, Institute of Biomedical & Health Science, Hiroshima University, 1-2-3 Kasumi Minami-ku, Hiroshima, Japan

## Abstract

**Introduction:**

The important functions of the meniscus are shock absorption, passive stabilization and load transmission of the knee. Because of the avascularity of two-thirds of the meniscal center region, the treatment of tears in this area is hard. Recently, microRNAs have been proven to play an important role in the pathogenesis of diseases. We focused on microRNA (miR)-210, which plays a wide spectrum of roles comprising mitochondrial metabolism, angiogenesis, DNA repair and cell survival. This study aimed to investigate the effect of intra-articular injection of synthetic miR-210 on the injured meniscus in the avascular zone.

**Methods:**

The middle segments of the medial meniscus of Spraque Dawley rats were incised longitudinally with a scalpel. An intra-articular injection of double-stranded (ds) miR-210 (for control group using control dsRNA) with atelocollagen was administered immediately after injury. Four weeks and 12 weeks after the injection, we conducted a histologic evaluation, immunohistochemical evaluation and Real-time PCR analysis. *In vitro*, the inner meniscus and synovial cells were isolated from rat knee joint, and were transfected with ds miR-210 or control dsRNA. Real-time PCR and immunohistochemical evaluations were performed.

**Results:**

Twenty-four hours after the injection, FAM (Fluorescein amidite) labeled miR-210 was observed in the cells around the injured site. Four weeks after the injection, the injured site of the miR-210 group was filled with repaired tissue while that of the control was not repaired. In gene expression analysis of the meniscus, the expression of miR-210, Collagen type 2 alpha 1 (Col2a1), Vascular endothelial growth factor (VEGF), and Fibroblast growth factor-2 (FGF2) in the miR-210 group was significantly higher than that in the control. At 12 weeks, the intra-articular injection of miR-210 had healed the injured site of the meniscus and had prevented articular cartilage degeneration. *In vitro*, miR-210 upregulated Col2a1 expression in the meniscus cells and VEGF and FGF2 expression in the synovial cells.

**Conclusions:**

An intra-articular injection of ds miR-210 was effective in the healing of the damaged white zone meniscus through promotion of the collagen type 2 production from meniscus cells and through upregulated of VEGF and FGF2 from synovial cells.

## Introduction

The meniscus is an important tissue for the functioning of the knee joint, and plays a crucial role in the knee load motion, stability and shock absorption of the knee. The medial and lateral menisci are C-shaped fibrocartilaginous wedges located between the femoral condyles and the tibial plateau [1,2]. In the human meniscus, the outer 10 to 20% of the meniscus is supplied by a perimeniscal capillary plexus, but 70 to 80% of the inner meniscus forms the avascular region [3,4]. Damaged meniscus produces pain, articular cartilage injury and functional loss of the knee joint, which is recognized as one of the key factors of osteoarthritis (OA) of the knee, and explains the real need for effective treatment [5]. Several studies have shown that tears in the avascular area do not heal successfully by suture repair alone [6,7]. Failure rates after attempted surgical repair remain high, ranging from 24 to 50% for isolated meniscal tears [8]. This high failure is due to the fact that the inner two-thirds of the menisci are avascular, and avascularity is considered one of the most important factors contributing to the poor healing potential of this region [3,9]. Such unsatisfactory outcomes have stimulated the development of a variety of techniques designed to augment repairs with an additional biological stimulus through synovial rasping, trephination or application of fibrin clot or platelet-rich plasma [10-13]. Newer techniques with increasing cost and complexity are also being tested in animal models, including local application of exogenous growth factors or stem cells [14]. Recent literature has suggested that synovial tissue-derived mesenchymal stem cells may have the potential to aid in healing and regeneration of cartilage injuries, such as those involving the meniscus [15,16]. More effective novel strategies for meniscal injury should be developed.

microRNAs (miRNAs) are endogenous noncoding small RNAs of approximately 22 nucleotides in length [17]. miRNAs are a new gene group that combines mRNA of the target gene and controls gene expression by inhibition of translation or degradation of mRNA [17,18]. The biological relevance of miRNAs has been investigated in physiological and pathological conditions, revealing their involvement in the fine tuning of biological events, such as cell proliferation, differentiation and cell death [17,18]. miRNAs have been shown to play an important role in the pathogenesis of human diseases [19-21]. Several therapeutic trials have examined the regulation of endogenous miRNAs that are related to disease pathogenesis through the *in vivo* administration of specific antisense oligoribonucleotides or double-stranded (ds) miRNAs [22-24].

Several groups have reported recently that miR-210 is a key player in angiogenesis in response hypoxia [25,26]. Overexpression of miR-210 has also been reported to stimulate the formation of capillary-like structures *in vitro* in normoxic conditions as well as vascular endothelial growth factor (VEGF)-driven cell migration [25-27]. Such angiogenesis is well known as one of the key factors for tissue repair, and promoting angiogenesis during the initiation phase of tissue repair could accelerate tissue healing [10,28,29]. Shoji and colleagues demonstrated that intra-articular injection of synthetic miR-210 could accelerate anterior cruciate ligament healing in the rat model via enhancement angiogenesis [30]. In their study, VEGF and fibroblast growth factor (FGF)-2 were upregulated in the injured ligament by administration of miR-210, which also contributes to acceleration of ligament healing. Therefore, we hypothesized that intra-articular injection of synthetic miR-210 could enhance the injured meniscal healing process. There have been no reports about intra-articular injection of miRNA for the repair of meniscal injury previously, so the purpose of the present study was to investigate the effect of intra-articular injection of synthetic miR-210 on injured meniscal healing in a rat model.

## Materials and methods

All procedures were performed according to the Guidelines for Animal Experimentation, Hiroshima University, and with the approval the Committee of Research Facilities for Laboratory Animal Sciences, Graduate School of Biomedical Sciences, Hiroshima University.

### Animals

Twelve-week-old male Sprague–Dawley rats (Charles River Laboratories Japan, Tokyo, Japan) that are skeletally mature were used in these experiments [16,31,32]. The rats were housed at the Laboratory Animal Center of Hiroshima University under standard diurnal conditions of light/dark, were fed a standard commercial diet and were given tap water *ad libitum.*

### Preparation of double-stranded miR-210 and small interfering RNA–atelocollagen complex

We used ds miR-210 that was designed for intra-articular injection in the experimental group (sequences 66-CUG-UGC-GUG-UGA-CAG-CGG-CUG-A-87 and 87-AGC-CCC-UGC-CCA-CCG-CAC-ACU-G-66 labeled with fluorescein amidite; B-Bridge International, Mountain View, CA, USA). dsRNA molecules with no specific function were also prepared for use as a control group (sequences 5-ATC-CGC-GCG-ATA-GTA-CGT-A-3 and 3-overhang dTdT/dTdT (sense/antisense) small interfering RNA negative control; B-Bridge International). Atelocollagen is a solution of highly purified collagen type 1 isolated from calf dermis by pepsin treatment (Koken, Tokyo, Japan). The ds miR-210 and atelocollagen complex was prepared by mixing an equal volume of atelocollagen (in phosphate-buffered saline, pH 7.4) and dsRNA solution (20 μg/15 μl) and mixing by rotation at 4°C for 20 minutes. The control dsRNA and atelocollagen complex was prepared identically. The miRNA/atelocollagen complexes were prepared immediately before injection.

### Surgical procedure

Fifty-six of the rats were anesthetized with an intraperitoneal injection of pentobarbital sodium (40 mg/kg). The right knee was exposed using the medial parapatellar approach with the patella laterally dislocated, and the medial meniscus was identified at the knee joint in full flexion. At the middle segment of each of the medial meniscus, we made a full-thickness longitudinal tear 2 mm long in the avascular zone (white–white zone) using a scalpel. The capsule and skin were then closed. An intra-articular injection of dsRNA (total volume 30 μl) was administered through the patellar tendon into the right knee joints using an insulin syringe with 29 G needle (Becton, Dickinson and Company, Franklin Lakes, NJ, USA). The rats were allowed unrestricted weight-bearing and motion of their knees.

### Histologic and immunofluorescence analyses

At 4 weeks after injection, the medial meniscus was harvested and a frozen section was prepared. Frozen serial sections of 6 μm thickness were derived from the horizontal plane through the defect and prepared for histological analysis (*n* = 6 for each group). Hematoxylin and eosin staining and safranin O staining were performed. All specimens were given a histological score between 0 and 3 according to the following grading scale: 0 points, no noticeable reaction at all; 1 point, no bridge linking the two components; 2 points, connective tissue between the two components; 3 points, explants, which had fibrous continuity between both sides of the gap [33]. All specimens were evaluated by three of our colleagues (YK, TN and TS) without knowledge of the treatment received (blinded evaluation). At 12 weeks after intra-articular injection, the knee joint was harvested and fixed with 4.0% paraformaldehyde at 4°C for 24 hours. The samples were then decalcified in 0.5 M ethylenediaminetetraacetic acid (pH 7.5), and embedded in paraffin. The sections were prepared at a thickness of 6 μm and stained with safranin O staining (*n* = 6 for each group). Three independent assessors (YK, TN and TS) graded each section using a modified Mankin scale for evaluation of cartilage degeneration [34].

For fluorescence microscopy of fluorescein amidite-labeled miRNA-210 in meniscus samples, 6 μm serial sections were mounted on saline-coated glass slides, air dried and fixed with 4.0% paraformaldehyde at 4°C for 5 minutes. 4′,6-Diamidino-2-phenylindole solution was then applied for 5 minutes for nuclear staining.

### Isolation of meniscus and synovial cells

*In vitro*, the inner meniscus cells and synovial cells were isolated in the rat knee joint from each donor (*n* = 6 for each group). After the rat’s medial meniscus was harvested, the inner portion of the meniscus (cut from the midpoint of the meniscus) was prepared. Inner meniscus cells were isolated by collagenase treatment [35]. Attached cells were used as inner meniscus cells between passages 2 and 4. Synovial cells were isolated by collagenase treatment and cultured. Synovial cells between passages 3 and 6 were used. ds miR-210 or ds negative control RNA were transfected into the meniscus or synovial cells using Lipofectamine RNAiMax (Invitrogen, Carlsbad, CA USA) according to the manufacturer’s protocol. Cultures were maintained in Dulbecco’s modified Eagle’s medium (Wako, Osaka, Japan) containing 10% fetal bovine serum, 50 IU/ml penicillin and 50 μg/ml streptomycin for 1 week.

### Transfection of double-stranded RNA *in vitro*

Isolated cells were maintained in Dulbecco’s modified Eagle’s medium with 10% fetal bovine serum (Life Technologies, Grand Island, NY, USA) and 1% antibiotic–antimycotic solution (Nacalai Tesque, Kyoto, Japan). Inner meniscus cells (5 × 10^4^) or synovial cells (5 × 10^4^) were seeded into the wells of 12-well plates and incubated for between 3 and 24 hours. The cells were transfected with miR-210 (20 nM) or silencer negative control RNA (20 nM; Life Technologies) using Lipofectamine RNAiMAX Transfection Reagent (Life Technologies).

### Real-time polymerase chain reaction

For PCR analysis, a 2 mm × 2 mm piece of meniscus including the injured site was resected from the control and miR-210 groups. In the normal group, the same size piece of medial meniscus at the same site was harvested from 12-week-old Sprague–Dawley rats (*n* = 5 for each group). Total RNA was isolated from repaired tissue of the meniscus *in vivo* and *in vitro* using TRIzol (Life Technologies). Complementary DNA was synthesized using Ready to Go You-Prime First-Strand Beads (GE Healthcare, Chalfont, UK) with total RNA (1 μg) and oligo(dT) primers. For miRNA expression analysis, reverse transcriptase reactions of mature miRNAs contained a sample of total RNA, 50 nM stem-loop reverse transcriptase primer, 10 × reverse transcriptase buffer, 100 mM each dNTPs, 50 U/μl MultiScribe reverse transcriptase and 20 U/μl RNase inhibitor. The 15 μl reactions were incubated in a thermocycler (BioRad, Hercules, CA, USA). Real-time quantitative PCR was carried out using TaqMan Gene Expression Assay probes for rno-miR-210, snoRNA-135, VEGF, collagen type 1 alpha 1 (Col1a1), FGF2 and ACTB. The expression levels for each gene were assessed relative to the expression of snoRNA-135 for miR-210, and relative to ACTB for other genes. A threshold cycle was observed in the exponential phase of amplification, and quantification of relative expression levels was performed using standard curves for the target genes and the endogenous control. Geometric means were used to calculate the delta-delta C_T_ values and were expressed as 2^–ΔΔCT^. The value of each control sample was set at 1 and used to calculate the fold-change of target genes.

### Immunohistochemical analysis

For immunofluorescence staining, 6 μm serial sections and meniscus and synovial cells fixed with 4.0% paraformaldehyde were prepared. After blocking by horse serum (Vector Laboratories, Burlingame, CA, USA), specimens were immediately stained with rabbit polyclonal anti-VEGF or anti-basic FGF antibody (concentration 1:100; Abcam, Cambridge, MA, USA), Ki67 antibody (concentration 1:100; Novus, Littleton, Colorado, USA), goat polyclonal anti-collagen type 1 antibody (concentration 1:50; Santa Cruz Biotechnology, Santa Cruz, CA, USA), mouse polyclonal anti-collagen type 2 antibody (concentration 1:100; Abcam) or fluorescein-labeled GSL I-isolectin B4 (concentration 1:100; Vector Laboratories). The secondary antibodies used were Alexa Fluor 488-conjugated or Alexa Fluor 568-conjugated goat anti-rabbit IgG for VEGF-A, Ki67 and FGF2, Alexa Fluor 488-conjugated or Alexa Fluor 568-conjugated rabbit anti-goat IgG for collagen type 1, and Alexa Fluor 488-conjugated or Alexa Fluor 568-conjugated goat anti-mouse IgG for collagen type 2 (concentration 1:500; all from Molecular Probes/Invitrogen, Carlsbad, CA, USA). 4′,6-diamidino-2-phenylindole solution was applied for 5 minutes to detect nuclear staining.

For each section, the number of Ki67-positive cells and the number of all cells were counted at × 40 magnification, and the ratio of Ki67-positive cells to all cells was calculated (*n* = 5 for each group). The number of blood vessels that were identified as positive isolectin B4, the marker for rat endothelial cells, was counted at × 40 magnification (*n* = 5 for each group). Five sections were randomly evaluated in each rat for counting cells and blood vessels.

### Statistical analysis

All data were expressed as the mean ± standard deviation. For the statistical analysis, one-way analysis of variance followed by Tukey’s *post hoc* analysis was used for the detection of differences between three groups, and the Mann–Whitney *U* test was used for the detection of differences between two groups. *P* < 0.05 was considered statistically significant.

## Results

### Intra-articular injection of miR-210 can accelerate meniscal healing

**Figure 1:**
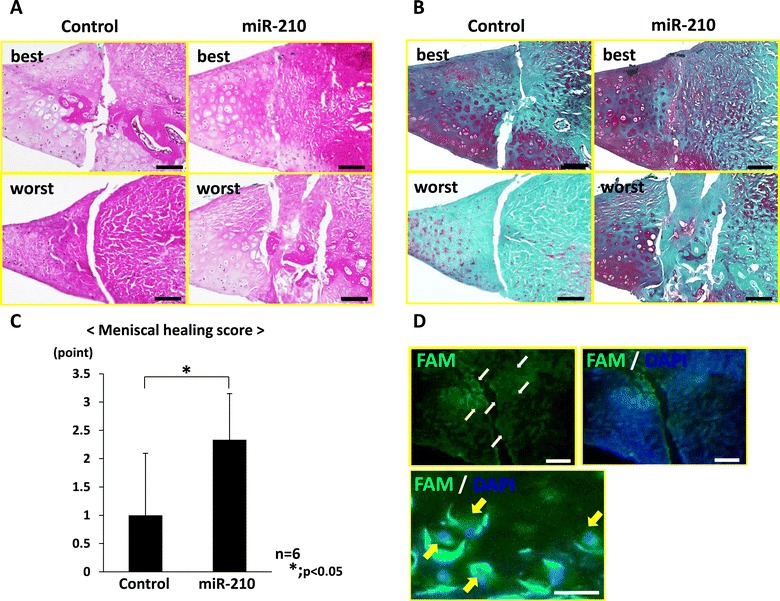
**Histological findings of the meniscus at 4 weeks. (A)** Hematoxylin and eosin staining. **(B)** Safranin O staining: the injured site of the miR-210 group was filled with repaired tissue stained with safranin O, while that of the control group was not repaired. Upper panels, best result; lower panels, worst result (bar = 100 μm). **(C)** Histological score of the miR-210 group (2.3 ± 0.8 points) was significantly higher than that of the control group (1.0 ± 1.0 point) (*P < 0.05, *n* = 6 for each group). **(D)** Detection of fluorescein amidite (FAM)-labeled double-stranded (ds) miR-210: distribution of green fluorescence was observed in the meniscus around the injured site 24 hours after injection of the FAM-labeled ds miR-210/atelocollagen complex into the joint (bar = 100 μm). Expression of green fluorescence was observed in the cytoplasm of cells in high-power views (bar = 25 μm). Arrows, FAM-labeled ds miR-210/atelocollagen complex. DAPI, 4′,6-diamidino-2-phenylindole.

The injured site of the miR-210 group was filled with repaired tissue stained with safranin O, while that of the control group was not repaired. In the miR-210 group, one-half of the rats showed complete meniscal healing, and the remaining rats showed high or intermediate healing. In the control group, no rat showed complete meniscal healing, and one-half of the rats showed no meniscal healing. The histological score of the miR-210 group (2.3 ± 0.8 points) was significantly higher than that of the control group (1.0 ± 1.0 points) (*P* < 0.05, *n* = 6 for each group) (Figure 1A,B,C). Fluorescein amidite-labeled ds miR-210/atelocollagen complex was taken in cells of the whole meniscus after intra-articular injection, and was observed in the cells around the injured site in particular (Figure 1D).

### Intra-articular injection of miR-210 prevents articular cartilage degeneration

**Figure 2:**
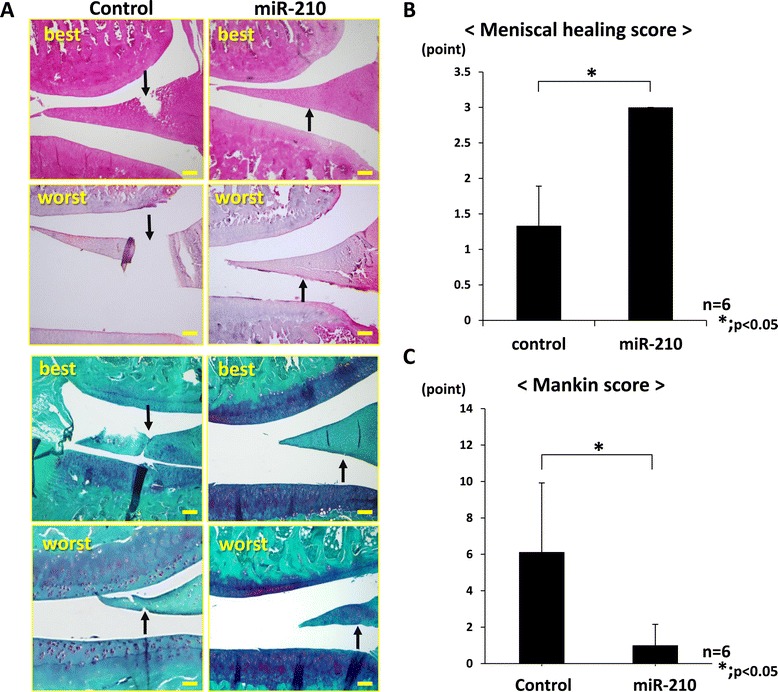
**Histological findings of the knee joint at 12 weeks. (A)** Hematoxylin and eosin staining and safranin O staining: the injured site in the miR-210 group was morphologically healed whereas the tear in the meniscus still remained in the control group (bar = 100 μm). Safranin O staining revealed that the degeneration of articular cartilage in the miR-210 group was lower than that of the control group. Arrow, injured site. Upper panels, best result; lower panels, worst result. **(B)** Meniscal healing score in the miR-210 group (3.0 ± 0.0 points) was significantly higher than in the control group (1.3 ± 0.6 points) (*P < 0.05, *n* = 6 for each group). Degenerative change of cartilage in the control group was more advanced than in the miR-210 group. **(C)** Mankin score in the miR-210 group (1.0 ± 0.4 points) was significantly lower than in the control group (5.6 ± 1.4 points), showing a significant difference between the two groups (*P < 0.05, *n* = 6 for each group). Intra-articular injection of miR-210 prevented articular cartilage degeneration.

Macroscopic findings showed that the injured site in the miR-210 group was morphologically healed, whereas the tear in the meniscus still remained in the control group at 12 weeks after the intra-articular injection. There was no obvious synovitis in both groups. In the control group, OA changes such as fibrillation, irregularity, erosion and loss of cartilage surface were observed. Histological findings for six out of six rats in the miR-210 group but only for two out of six rats in the control group showed complete meniscal healing. The meniscal healing score of the miR-210 group was significantly higher than that of the control group (*P* < 0.05, *n* = 6 for each group) (Figure 2A). Safranin O staining revealed more severe degenerative change of the cartilage in the control group compared with the miR-210 group. In the control group, OA changes such as fissure of cartilage surface, loss of outer layer cartilage cells and reduction of stainability were observed (Figure 2B). Regarding the Mankin score, there was a significant difference between the two groups (*P* < 0.05, *n* = 6 for each group) (Figure 2C).

### Gene expression analysis of the meniscus

**Figure 3:**
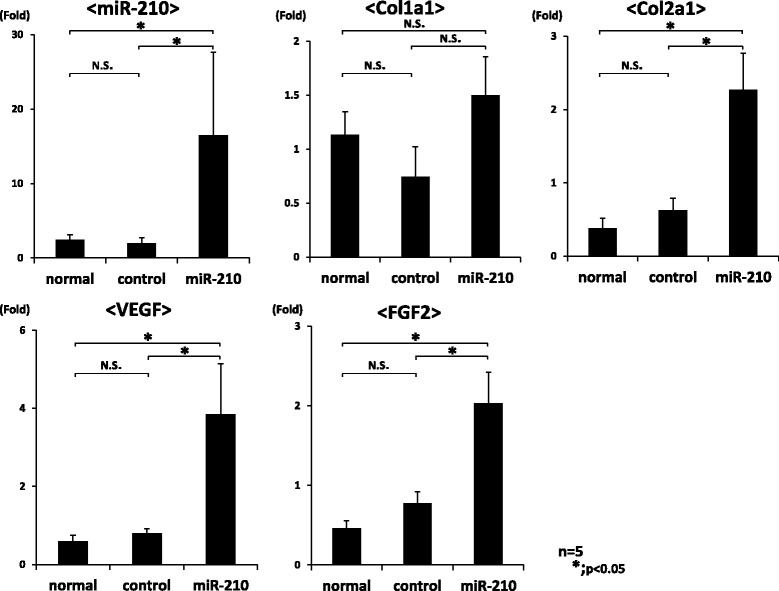
**Gene expression analysis by real-time PCR in the meniscus at 4 weeks following intraarticular injection.** Expression of mature miR-210, collagen type 1 alpha 1 (Col1a1), collagen type 2 alpha 1 (Col2a1), vascular endothelial growth factor (VEGF) and fibroblast growth factor-2 (FGF2) was examined using real-time PCR. Expression of miR-210, Col2a1, VEGF and FGF2 in the miR-210 group was significantly higher than that in the control and normal groups (*P < 0.05). There was no significant difference in miR-210, Col2a1, VEGF and FGF2 expression between the control and normal groups (*P < 0.05, N.S. (no significant difference), *n* = 5 for each group).

For gene expression analysis in the meniscus, mature miR-210, Col1a1, collagen type 2 alpha 1 (Col2a1), VEGF and FGF2 expression was examined using real-time PCR. The expression of miR-210, Col2a1, VEGF and FGF2 in the miR-210 group was significantly higher than that in the control and normal groups (*P* < 0.05, *n* = 5 for each group) (Figure 3). There is no significant difference in expression between the control and normal groups. For Col1a1 expression, there was no significant difference between each group.

**Figure 4:**
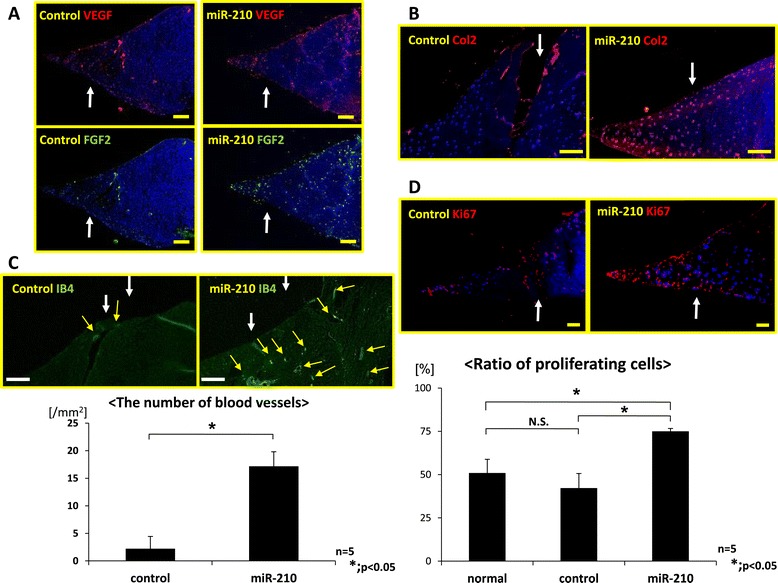
**Immunohistochemistry of meniscus at 4 weeks after intra-articular injection. (A)** Immunohistochemistry of vascular endothelial growth factor (VEGF; upper) and fibroblast growth factor-2 (FGF2; lower). VEGF and FGF2 expressed intensely on the surface of the meniscus, around the injured site and in the red zone in the miR-210 group compared with the control group. Arrow, injured site. Bar = 100 μm. **(B)** Immunohistochemistry of type 2 collagen. Type 2 collagen expression was observed around the injured site of the meniscus in the miR-210 group compared with the control group. Arrow, injured site. Bar = 100 μm. **(C)** Isolectin B4 staining and the number of blood vessels. Newly formed vessels were observed around the injured site in the miR-210 group, while little blood vessels were observed in the control. White arrow, injured site; yellow arrow, blood vessels. Bar = 100 μm. Number of blood vessels in the miR-210 group was significantly higher than that in the control group (*P < 0.05, *n* = 5 for each group). **(D)** Immunohistochemistry of Ki67 and the ration of proliferative cells. In the miR-210 group, many proliferative cells were observed around the injured site. Arrow, injured site. Proliferative cells in the miR-210 group were significantly higher than that in the control group (*P < 0.05, N.S. (no significant difference), *n* = 5 for each group).

In immunohistochemistry, VEGF and FGF2 intensely expressed on the surface of the meniscus, around the injured site and in the red zone in the miR-210 group compared with the control group (Figure 4A). Type 2 collagen expression was observed around the injured site of the meniscus in the miR-210 group, while its expression was sparse in the control (Figure 4B). In the miR-210 group, newly formed vessels were observed around the injured site, while few blood vessels were observed in the control group. The number of blood vessels in the miR-210 group was significantly higher than that in the control group (*P* < 0.05, *n* = 5 for each group) (Figure 4C). In the miR-210 group, many proliferative cells that had an immunoreactivity of Ki67 were observed around the injured site. Proliferative cells in the miR-210 group were significantly higher than those in the control group (*P* < 0.05, *n* = 5 for each group) (Figure 4D).

### miR-210 upregulated Col2a1 expression in the inner meniscus cells and VEGF and FGF2 expression in the synovial cells

**Figure 5:**
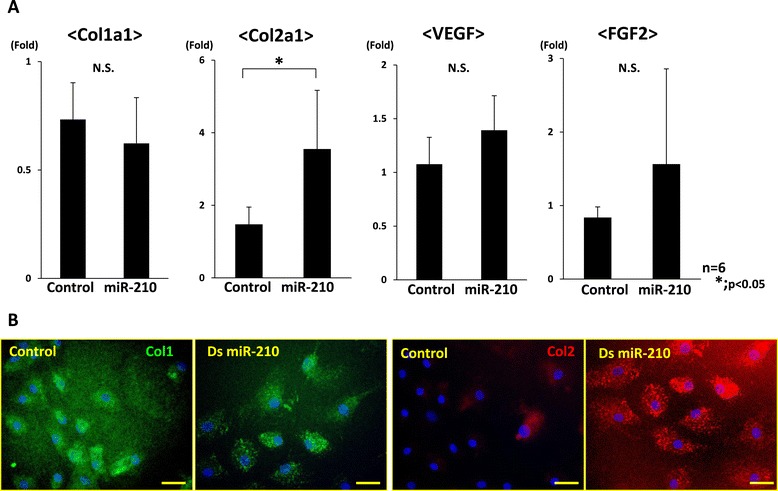
**Gene expression analyses in inner meniscus cells after overexpression of miR-210. (A)** Real-time PCR analysis of collagen type 1 alpha 1 (Col1a1), collagen type 2 alpha 1 (Col2a1), vascular endothelial growth factor (VEGF) and fibroblast growth factor-2 (FGF2) at 7 days after *in vitro* transfection of inner meniscus cells. Expression of only Col2a1 was significantly higher than that in the control group (*P < 0.05, N.S. (no significant difference), *n* = 6 for each group). **(B)** Immunohistochemical analysis indicates that collagen type 2 was highly expressed in the miR-210 group (bar = 100 μm). These results demonstrate that miR-210 could enhance the expression of collagen type 2 in the meniscus cells. ds, double stranded.

**Figure 6:**
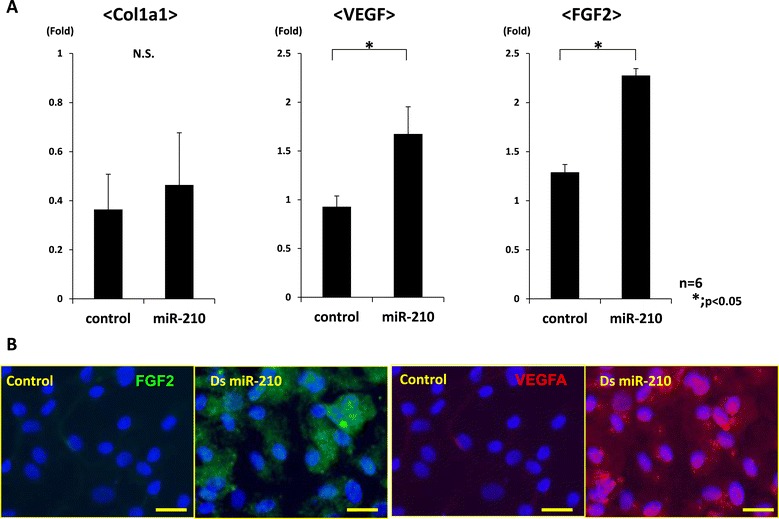
**Gene expression analyses in synovial cells after overexpression of miR-210. (A)** Real-time PCR analysis of collagen type 1 alpha 1 (Col1a1), collagen type 2 alpha 1 (Col2a1), vascular endothelial growth factor (VEGF) and fibroblast growth factor-2 (FGF2) at 7 days after *in vitro* transfection of synovial cells. Expression of Col2a1 was not detected in both groups. Expression of VEGF and FGF2 was significantly higher than in the control group (*P < 0.05, N.S. (no significant difference), *n* = 6 for each group). **(B)** Immunohistochemical analysis indicates that VEGF and FGF2 were highly expressed in the miR-210 group (bar = 100 μm). These results demonstrated that miR-210 could enhance the expression of VEGF and FGF2 expression in synovial cells. ds, double stranded.

Real-time PCR revealed that Col2a1 expression was significantly upregulated in the inner meniscus cells, and immunocytochemistry also demonstrated that miR-210 could enhance the expression of collagen type 2 in the meniscus cells (*P* < 0.05, *n* = 6 for each group) (Figure 5A,B). On the other hand, the expression of VEGF and FGF2 was significantly upregulated in the synovial cells (*P* < 0.05, *n* = 6 for each group) (Figure 6A). Immunocytochemistry indicated that VEGF and FGF2 expression were enhanced in the synovial cells (Figure 6B).

## Discussion

Generally, partial or overall meniscectomy is performed to treat injured meniscus, especially in the avascular region. However, this results in reduced articular stability and exposes the articular cartilage surface to higher contact stress. The meniscus is dimorphic tissue, and it is very likely that an injury located in the outer one-third of the meniscus (red zone) is repaired more effectively than an injury in another area, thanks to the red zone’s dense blood vessels. Avascularity of the inner two-thirds of the meniscus (white zone) is associated with limited healing potential of this zone. The ideal treatment for meniscal injury is considered to promote both angiogenesis from the perimeniscal capillary plexus and upregulation of extracellular matrix in both inner and outer meniscus regions.

Recently, miRNAs have attracted immense attention because of their pivotal role in human disease, and they have been proposed as potential new therapeutic targets. Several therapeutic trials to regulate miRNA *in vivo* have been undertaken [24,30]. It has been reported that miR-210 is a crucial molecule of the endothelial cell response to hypoxia and that the targets of miR-210 are ephrin-A3, E2F3, MN, ACVR1B, NPTX1, RAD52, CASP8AP2, FGFRL1 and HOXA-1/9, which have important functions in cell survival, migration and differentiation [25,36-43]. Several papers have reported the involvement of miR-210 in angiogenesis. Hu and colleagues demonstrated that local injection of ds miR-210 generated in a minicircle vector into the myocardium can improve angiogenesis, inhibit apoptosis and improve cardiac function in mice [44]. Shoji and colleagues demonstrated that intra-articular injection of ds miR-210 can promote the healing of partially torn anterior cruciate ligaments through enhancement of angiogenesis [30]. In their research, collagen type 1 expression was upregulated in the injured ligament after the administration of miR-210, although it was unclear whether collagen type 1 production occurred as a direct or indirect effect of miR-210. In our current study, the production of collagen type 2 was enhanced by the administration of miR-210 *in vivo*, and notably this phenomenon could be observed in isolated cultured meniscus cells. This indicated the direct effect of miR-210 on upregulation of collagen type 2 in inner meniscus cells. The main composition of the extracellular matrix of the meniscus is collagen type 1, but in the inner region of the meniscus, which is composed of avascular tissue, extracellular matrix contains collagen type 2 and a greater number of chondrocytic morphology cells than in the outer region of the meniscus [45,46]. Several studies have attempted upregulation of collagen type 2 in meniscus cells using cytokines, mechanical stress and cells [10,14,47,48].

The effect of growth factors on stimulating angiogenesis from perimeniscal capillaries to enhance meniscal healing has been the focus of several studies [4,9,49,50]. VEGF and FGF2 are well known as potent angiogenetic factors. FGF2 is recognized to stimulate fibroblast proliferation, angiogenesis and enhancement of collagen synthesis [51]. Narita and colleagues demonstrated that FGF2 can stimulate the proliferation of meniscus cells in an organ culture model [52]. VEGF is able to induce endothelial cell migration, and several studies demonstrated attempts to promote meniscal healing by VEGF, but they did not achieve sufficient vessel formation and meniscal healing [9,49]. Administration of single growth factor is not always successful for tissue regeneration. Synovium plays an important role in meniscal healing, and it is reported to produce several growth factors to enhance meniscal healing [10,53,54]. In our results, high expression of VEGF and FGF2 was observed *in vivo* after intra-articular injection of miR-210. To examine which cell type in the meniscal lesion contributed to the meniscal healing by the miR-210 injection, overexpression of miR-210 was conducted in isolated inner meniscus cells or synovial cells in rats *in vitro*. VEGF and FGF2 expression in synovial cells were upregulated by overexpression of miR-210. Immunohistochemistry of meniscus at 4 weeks indicated that VEGF and FGF2 were intensely expressed not only on the injured site but also on the surface of the meniscus, which might mean induction of synovium to the injured site along the surface of the meniscus. miR-210 is recognized to have the potent function of angiogenesis, and in this study intra-articular injection of ds miR-210 could enhance the angiogenesis around the injured site of the meniscus. As for the cell proliferation, miR-210 could promote cell proliferation in the meniscus. Moreover, miR-210 could directly promote collagen synthesis in inner region meniscus cells. It has been reported that factors affecting tissue healing in the joint and a healing response include the biomechanical forces, the surrounding tissues, blood supply, nutrient delivery, synovial fluid and the supply of growth factors [10,28]. miR-210 delivery into the knee joint may therefore have several pleiotropic effects on several tissues including the meniscus itself, the synovium and surrounding tissues, in addition to the proangiogenic roles that were investigated here. Hundreds of miRNA target genes are predicted by computer analysis, which means that miRNA therapy has more multifactorial effects on tissue regeneration compared with single growth factor administration. Furthermore, unlike cell therapy, miRNA therapy does not require cells to be harvested and cultured.

The limitations of this study are as follows. First, we could not analyze the local or systemic adverse effects of an intra-articular injection. miR-210 plays a role in the induction of VEGF expression, which is recognized as harmful for articular cartilage. We have confirmed that intra-articular injection of ds miRNA is not able to be taken up by chondrocytes in normal cartilage (data not shown). However, there is the possibility that ds miRNA can be taken up by OA cartilage, which might promote cartilage degeneration. Nagata and colleagues demonstrated the systemic distribution of ds miRNA after intra-articular injection [55]. They showed that injected ds miR-15a, which induces cell apoptosis, was detected in the liver, but no apoptosis in the liver was observed. Second, the molecular mechanism is still unclear. Several reports link miR-210 to the pathogenesis of cancer, so further investigation including the validation of target genes is required.

## Conclusion

The present study demonstrates that intra-articular injection of ds miR-210 can promote the healing of damaged white zone meniscus through the promotion of collagen type 2 production in meniscus cells and through the enhancement of VEGF and FGF2 expression in synovial cells with angiogenesis and cell proliferation. Administration of miRNA would be expected to bring about more effects via regulation of many gene networks, which would be an advantage of miRNA therapy. The results of this study suggest that administration of synthetic miR-210 would be a meniscus treatment option, although the exact process and molecular mechanism of meniscal healing have not been fully elucidated. Intra-articular injection of ds miRNA *in vivo* is a potential new and exciting strategy for the future treatment of injured joints. However, to determine its competency as a therapeutic agent, more extensive study involving elucidation of the target genes and of the adjustment process is necessary.

## Abbreviations

Col1a1: collagen type 1 alpha 1
Col2a1: collagen type 2 alpha 1
ds: double-stranded
FGF: fibroblast growth factor
miR: microRNA
OA: osteoarthritis
VEGF: vascular endothelial growth factor
